# *In Silico* Evaluation of the Potential Antiarrhythmic Effect of Epigallocatechin-3-Gallate on Cardiac Channelopathies

**DOI:** 10.1155/2016/7861653

**Published:** 2016-11-02

**Authors:** Maroua Boukhabza, Jaouad El Hilaly, Nourdine Attiya, Ahmed El-Haidani, Younes Filali-Zegzouti, Driss Mazouzi, Mohamed-Yassine Amarouch

**Affiliations:** ^1^Materials, Natural Substances, Environment and Modeling Laboratory, Multidisciplinary Faculty of Taza, University Sidi Mohamed Ben Abdellah, Fez, Morocco; ^2^Biology, Environment & Health Team, Department of Biology, Faculty of Sciences and Techniques Errachidia, University of Moulay Ismaïl, Meknes, Morocco; ^3^Department of Life and Earth Sciences, Regional Institute of Education and Training Careers, Fez, Morocco; ^4^Team of Nutritional Physiology and Endocrine Pharmacology, Department of Biology, Faculty of Sciences and Techniques Errachidia, University of Moulay Ismaïl, Meknes, Morocco

## Abstract

Ion channels are transmembrane proteins that allow the passage of ions according to the direction of their electrochemical gradients. Mutations in more than 30 genes encoding ion channels have been associated with an increasingly wide range of inherited cardiac arrhythmias. In this line, ion channels become one of the most important molecular targets for several classes of drugs, including antiarrhythmics. Nevertheless, antiarrhythmic drugs are usually accompanied by some serious side effects. Thus, developing new approaches could offer added values to prevent and treat the episodes of arrhythmia. In this sense, green tea catechins seem to be a promising alternative because of the significant effect of Epigallocatechin-3-Gallate (E3G) on the electrocardiographic wave forms of guinea pig hearts. Thus, the aim of this study was to evaluate the benefits-risks balance of E3G consumption in the setting of ion channel mutations linked with aberrant cardiac excitability phenotypes. Two gain-of-function mutations, Na_v1.5_-p.R222Q and Na_v1.5_-p.I141V, which are linked with cardiac hyperexcitability phenotypes were studied. Computer simulations of action potentials (APs) show that 30 *μ*M E3G reduces and suppresses AP abnormalities characteristics of these phenotypes. These results suggest that E3G may have a beneficial effect in the setting of cardiac sodium channelopathies displaying a hyperexcitability phenotype.

## 1. Introduction

Ion channels are transmembrane proteins that allow the passage of ions according to the direction of their electrochemical gradients across cell membranes. They are pore-forming membrane proteins whose normal function is critical for several physiological processes in cells. In excitable cells, such as cardiac cells, the activity of these proteins maintains the resting membrane potential and generates action potentials that are essential for excitation-contraction coupling process.

Ion channel dysfunction is the principal pathophysiological mechanism underlying various inherited forms of arrhythmic disorders, also called channelopathies [1]. In cardiac cells, mutations in more than 30 genes encoding ion channels have been associated with an increasingly wide range of inherited cardiac arrhythmias [1]. Examples of genetic cardiac disorders include congenital ectopic Purkinje-related premature contractions (MEPPC) and exercise induced polymorphic ventricular tachycardia (EPVT) which have been linked to the presence of the Na_v1.5_-p.R222Q and Na_v1.5_-p.I141V mutations [2–4].

The ECG of the Na_v1.5_-p.R222Q carriers displayed atrial fibrillation, narrow junctional, and rare sinus beats competing with numerous premature ventricular contractions. The observed arrhythmia disappears under exercise [2, 3]. For Na_v1.5_-p.I141V carriers, the ECG is characterized by an increased sinus rate, atrial tachyarrhythmias, and an increased number of ventricular complexes during exercise [4]. On the molecular level, these mutations affect the biophysical properties of Na_v1.5_ by shifting its voltage dependence of steady state of activation towards more negative potentials and accelerating its activation and inactivation kinetics [2–6].

Ion channels become one of the most important molecular targets for several classes of drugs including antiarrhythmics and local anesthetic molecules [7]. In this sense, green tea flavonoids could offer a natural promising alternative. Indeed, Epigallocatechin-3-Gallate (E3G), as a major flavanol of green tea, has shown significant effects on the electrocardiographic wave forms in guinea pig. This compound has been demonstrated to exhibit inhibitory action on several cardiac ion channels [8].

Tea is manufactured from the dried leaves of* Camellia sinensis *in three basic forms of nonoxidized (green), semioxidized (oolong), and oxidized (black). Green tea is one of the most widely consumed beverages in North Africa and exhibits high content in polyphenolic flavanols known as catechins which may constitute up to 36% of the dry leaf weight [9, 10]. Catechins represent 80% to 90% of green tea total flavonoids, where epigallocatechin gallate appears to be the major predominant catechin (48–55%) followed by epigallocatechin (9–12%), epicatechin gallate (9–12%), epicatechin (5–7%), and a small proportion of catechin (0.3–0.6%) [11].

Most flavonoids affect vascular system insofar to normalize blood pressure by either inhibiting calcium channels or activating potassium channels or both. But contrary to the clear-cut pathophysiological benefit of flavonoids on vascular system, the impact of these compounds on cardiac channelopathies is yet somewhat unclear [12]. Previous investigation using patch clamp technique showed that flavonoids act as multichannel inhibitors, thereby triggering generally unexpected pharmacological effects. Therefore, the reported effects on cardiac ion channels of most flavonoids remain largely unknown whether they are anti- or proarrhythmic [12, 13]. In this sense, voltage gated sodium channel (VGSC) inhibition by polyphenols is well documented as cardioprotective and antiarrhythmic pathways. Catechins, like other polyphenols, share the common structural feature of one or more phenolic rings with several antiarrhythmic VGSC inhibitors such as lidocaine and mexiletine. These polyphenolic compounds may also inhibit peak and/or late *I*_Na_, leading to beneficial impact on the parameters associated with arrhythmias.

In this context, the aim of this study was to evaluate the benefits-risks balance of E3G effect on the setting of cardiac channelopathies.

## 2. Materials and Methods

### 2.1. Models

The action potentials were simulated using the updated mathematical model of the human atrial action potential of Maleckar-Greenstein-Trayanova-Giles (MGTG) [14], Stewart–Aslanidi–Noble–Noble–Boyett–Zhang (SANNBZ) Purkinje cell model [15], and Tusscher–Noble–Noble–Panfilov (TNNP) human ventricular cell models [16].

### 2.2. Formulation of Fast Sodium Current

In TNNP and SANNBZ models, the sodium current *I*_Na_ is represented according to a Hodgkin–Huxley formalism: *I*_Na_ = *g*_Na_ *m* ^3^ *hj*(*V*_m_ − *E*_Na_), where *g*_Na_ is the maximal conductance of *I*_Na_. *m* ^3^, *h*, and *j* are the activation gate, fast inactivation gate, and slow inactivation gate, respectively. *V*_m_ represents the membrane potential, and *E*_Na_ is the Nernst potential of sodium.

For the MGTG atrial model, the sodium current *I*_Na_ is represented according to the following equation:

(1) INa=PNam30.9h1+0.1h2Na+cVF2RTeV−ENaF/RT−1eVF/RT−1,

where *P*_Na_ represents the permeability to the sodium and *m* ^3^,* h*_1_, and *h*_2_ are the activation gate, fast inactivation gate, and slow inactivation gate, respectively. *V* represents the membrane potential, and *E*_Na_ is the Nernst potential of sodium.

### 2.3. Computer Modeling of Na_v1.5_-p.R222Q and Na_v1.5_-p.I141V Mutants

The same strategy was used for all models of the atrial, human Purkinje cells, and left-ventricular myocytes [15–18]. As reported by Mann et al., 2012, and Swan et al., 2014, [3, 4], the equations corresponding to the *I*_Na_ current were modified to reproduce the relative variation of biophysical properties of the sodium current due to the Na_v1.5_-p.R222Q and Na_v1.5_-p.I141V mutations.

The effects of the Na_v1.5_-p.R222Q and Na_v1.5_-p.I141V mutations were simulated as previously described by Mann et al., 2012, and Swan et al., 2014, for the ventricular and Purkinje models [3, 4]. *m*_*∞*_, *h*_*∞*_, *α*_*m*_, *β*_*m*_, and *β*_*h*_ were modified to reproduce the shift in the voltage dependencies of steady state of activation and inactivation and their kinetics. For the MGTG atrial model, *m*_*∞*_, *m* factor, *h*_*∞*_, and *h* factor were modified to reproduce the shift of the activation and inactivation curves as well as the changes observed in the sodium current kinetics. In all conditions, the heterozygous states were reproduced by the summation of half the WT current and half the mutant current.

### 2.4. Computer Modeling of Epigallocatechin-3-Gallate Effect on Ion Channels

For all models and conditions (WT and mutants), the effects of E3G on ion channels were reproduced based on the experimental work of Kang et al. [8].

Table 1 summarizes the modifications of the cardiac ion currents that were introduced in all models to match the experimental Data.

### 2.5. Conduction Velocity

Conduction velocity was investigated in fibers of MGTG, TNNP, and SANNBZ cell models (pacing rates: 1 Hz for the atrial and ventricular models and 2.5 Hz for the Purkinje model).  Table 3 summarizes the parameters used for the calculation of conduction velocity.

All simulations were performed by Myokit v.1.20.5 [19].

## 3. Results

### 3.1. Simulated Effect of E3G on the Electrical Activity of Cardiac Cells

**Figure 1:**
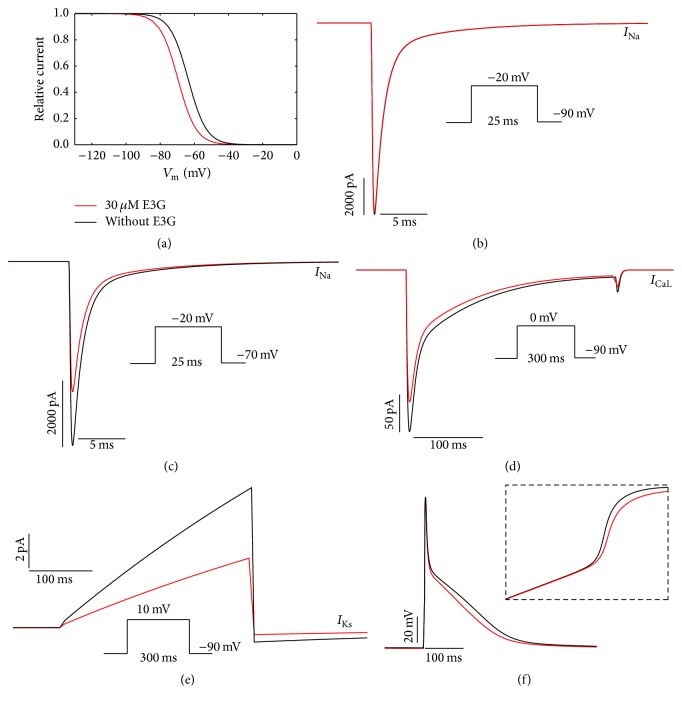
Simulated effects of 30 *μ*M E3G in the human atrial model (MGTG (Maleckar-Greenstein-Trayanova-Giles)). (a) Steady state inactivation curves (*h*_∞_) in the presence or absence of E3G. (b) and (c) Inhibitory effect of E3G on the sodium current at −90 mV and −70 mV resting potentials. (d) Inhibitory effect of E3G on the calcium current (20% inhibition). (e) Inhibitory effect of E3G on the *I*_Ks_ current (50% inhibition). (f) E3G effect on the atrial action potential (intensity of stimulus = 15 *μ*A/*μ*F, duration: 6 ms; inset, zoom of the rapid depolarization phase). For all panels, black lines: without E3G; red lines: 30 *μ*M E3G.

**Figure 2 fig2:**
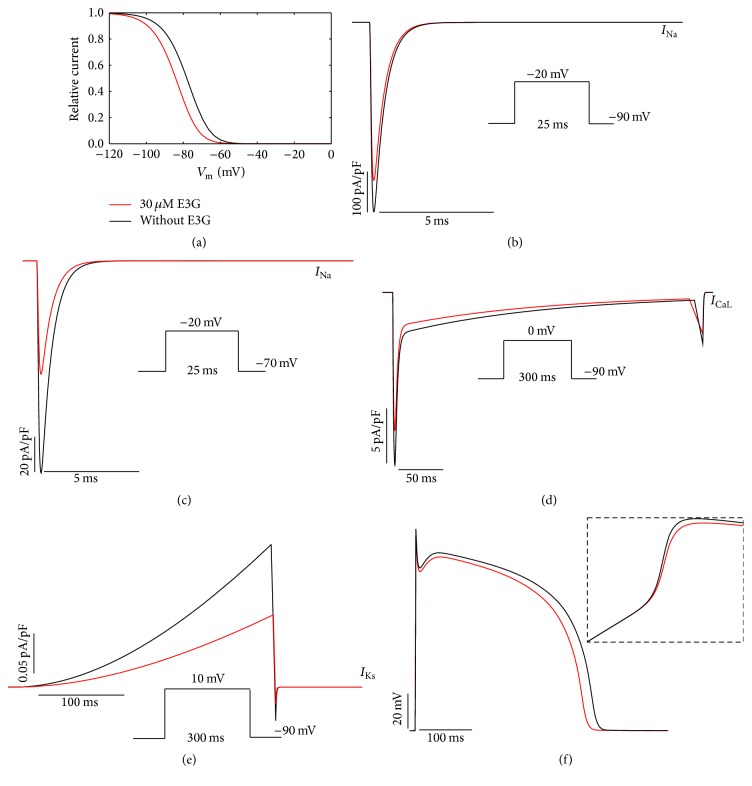
Simulated effects of 30 *μ*M E3G in the human ventricular model (TNNP [Tusscher–Noble–Noble–Panfilov]). (a) Steady state inactivation curves (*h*_∞_) in the presence or absence of E3G. (b) and (c) Inhibitory effect of E3G on the sodium current at −90 mV and −70 mV resting potentials. (d) Inhibitory effect of E3G on the calcium current (20% inhibition). (e) Inhibitory effect of E3G on the *I*_Ks_ current (50% inhibition). (f) E3G effect on the midmyocardial action potential (intensity of stimulus = 52 *μ*A/*μ*F, duration: 1 ms; inset, zoom of the rapid depolarization phase). For all panels, black lines: without E3G; red lines: 30 *μ*M E3G.

**Figure 3:**
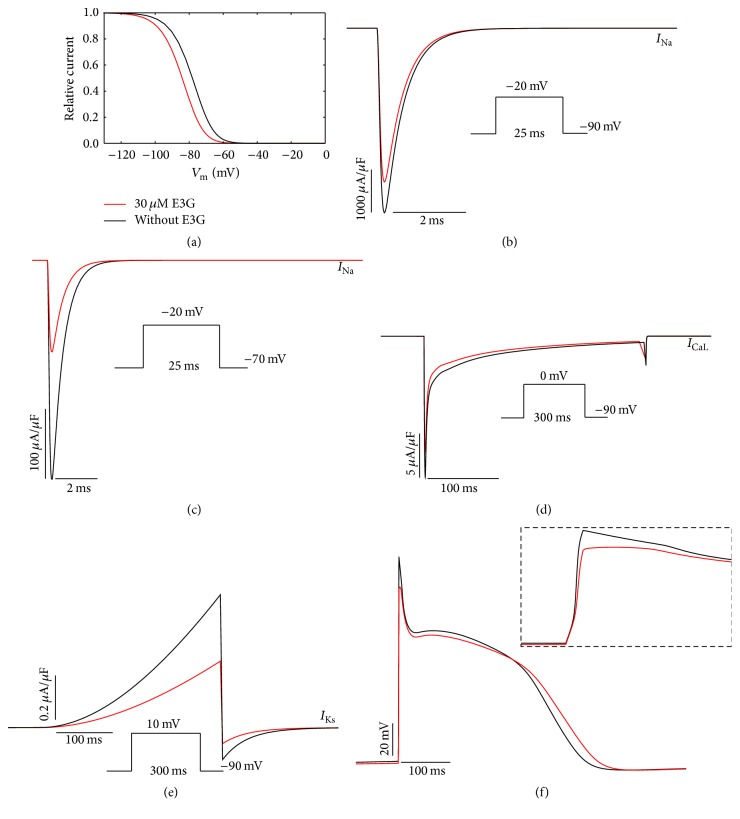
Simulated effects of 30 *μ*M E3G in the human Purkinje model (SANNBZ (Stewart–Aslanidi–Noble–Noble–Boyett–Zhang Purkinje cell model)). (a) Steady state inactivation curves (*h*_∞_) in the presence or absence of E3G. (b) and (c) Inhibitory effect of E3G on the sodium current at −90 mV and −70 mV resting potentials. (d) Inhibitory effect of E3G on the calcium current (20% inhibition). (e) Inhibitory effect of E3G on the *I*_Ks_ current (25% inhibition: dashed line; 50% inhibition: solid line). (f) E3G effect on the Purkinje action potential (intensity of stimulus = 52 *μ*A/*μ*F, duration: 1 ms; inset, zoom of the rapid depolarization phase). For all panels, black lines: without E3G; red lines: 30 *μ*M E3G.

To investigate the functional consequences of 30 *μ*M E3G on the electrical activity of atrial, Purkinje, and ventricular cells, we used MGTG, SANNBZ, and human epicardial, midmyocardial, and endocardial ventricular TNNP cell models. Using these models, the observed changes in cardiac ion channels function such as voltage dependencies and current amplitudes were implemented (see Section 2, Figures 1 [Fig fig2]–3).

In the *I*_Na_ formulations of MGTG, SANNBZ, and TNNP cell models, the effects of E3G on the sodium channel function were simulated by shifting the voltage dependence of the steady state equilibrium *h*_*∞*_ of *h* gate by −6 mV (Figures 1(a), 2(a), and 3(a)). The *m* and *j* gates were left unchanged.

The introduction of a negative shift in the inactivation curve, related to the presence of E3G, allowed us to reproduce the inhibitory effect of this compound on the sodium current amplitude for the ventricular and Purkinje cell models (Figures 2(b), 2(c), 3(b), and 3(c)). Indeed, as reported by Kang et al. [8], the inhibitory effect of E3G is higher at depolarized resting potentials. However, for the atrial cell model, we observed that 30 *μ*M E3G decreases the sodium current amplitude only when the resting potential is maintained at −70 mV (Figures 1(b) and 1(c)). There is no E3G effect when the resting potential is maintained at −90 mV. This is due to the biophysical properties of inactivation at basal condition. In fact, as shown in Figure 1(a), there is no difference in the sodium channel availability with or without E3G at −90 mV. Thus, an equal number of sodium channels were available in both conditions when the membrane was maintained at this potential.

Moreover, by using the same voltage protocols described by Kang et al., the inhibitory effect of 30 *μ*M E3G on *I*_CaL_ and *I*_Ks_ currents was reproduced via decreasing the amplitude of these currents to 80% and 50% of WT amplitude, respectively (Figures 1(d), 1(e), 2(d), 2(e), 3(d), and 3(e)). All used formulations are summarized in Tables 1 and 2 (see Section 2). Simulations were run for 60 s with a cycle length of 1 Hz to stabilize the model. Then, supplementary run was started for another 5 s and then the last AP of each supplementary run was analyzed.

The combined effects of 30 *μ*M E3G on cardiac ion channels slightly decreased the AP amplitudes and maximum upstroke velocities of atrial, Purkinje, and ventricular cells (Figures 1(f), 2(f), and 3(f)). In addition, the plateau phase of atrial AP was reduced (Figure 1(f)). However, E3G increased AP duration in Purkinje cell model (Figure 2(f)).

**Figure 4:**
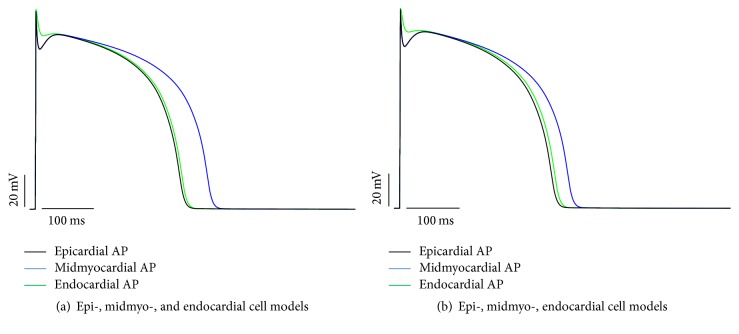
Repolarization dispersion across the ventricular wall. (a) Superimposition of the epi-, midmyo-, and endocardial action potentials (TNNP cells models) in absence of E3G. (b) Superimposition of the epi-, midmyo-, and endocardial action potentials in the presence of 30 *μ*M E3G.

For midmyocardial cells, E3G shortened the AP duration (Figure 3(f)). On the other hand, the superimposition of the epi-, midmyo-, and endocardial APs predicted a small decrease of the repolarization dispersion across the ventricular wall (Figure 4).

### 3.2. The p.R222Q and p.I141V Effects on Cardiac Excitability

**Figure 5:**
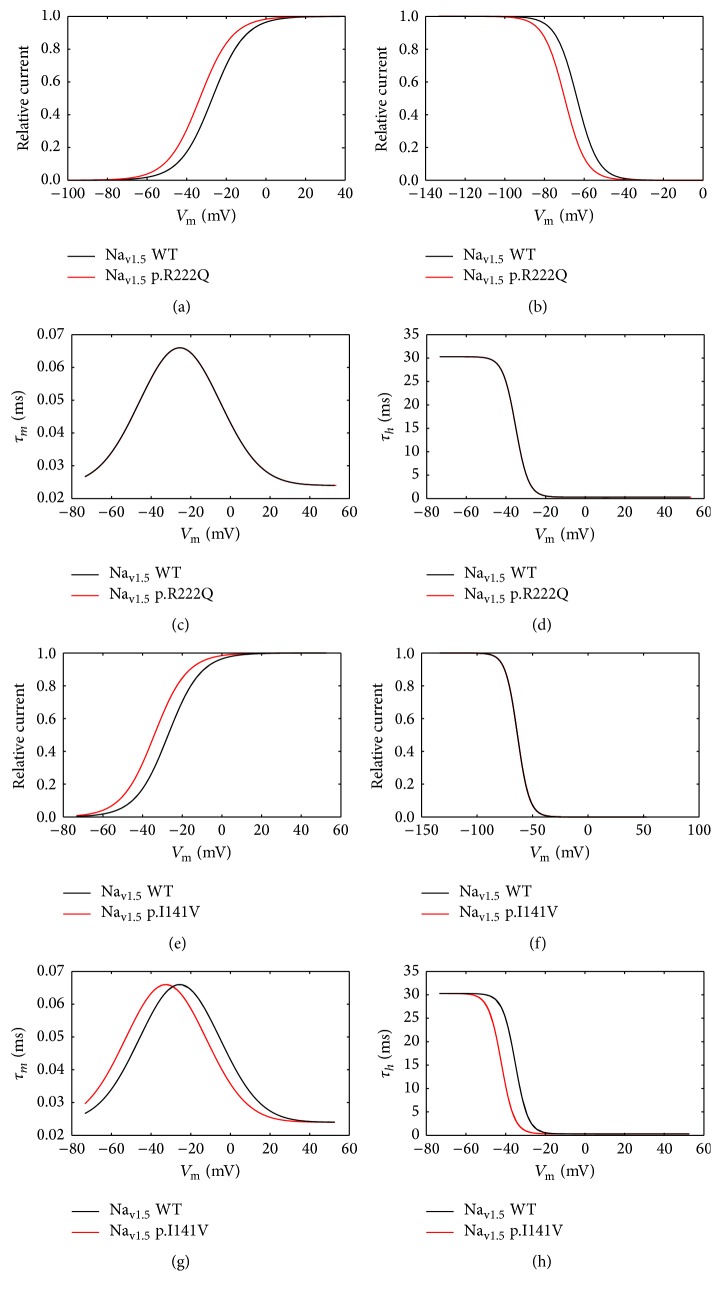
Effects of the p.R222Q and p.I141V mutations on *I*_Na_ properties in the human atrial cell model (MGTG cell model). (a) and (b) Effect of the p.R222Q mutation on the voltage dependence of steady state of activation and inactivation. (c) and (d) Effect of the p.R222Q mutation on the activation and inactivation kinetics. (e) and (f) Effect of the p.I141V mutation on the voltage dependence of steady state of activation and inactivation. (g) and (h) Effect of the p.I141V mutation on the activation and inactivation kinetics. For all panels, black lines: WT condition; red lines: mutant conditions.

**Figure 6:**
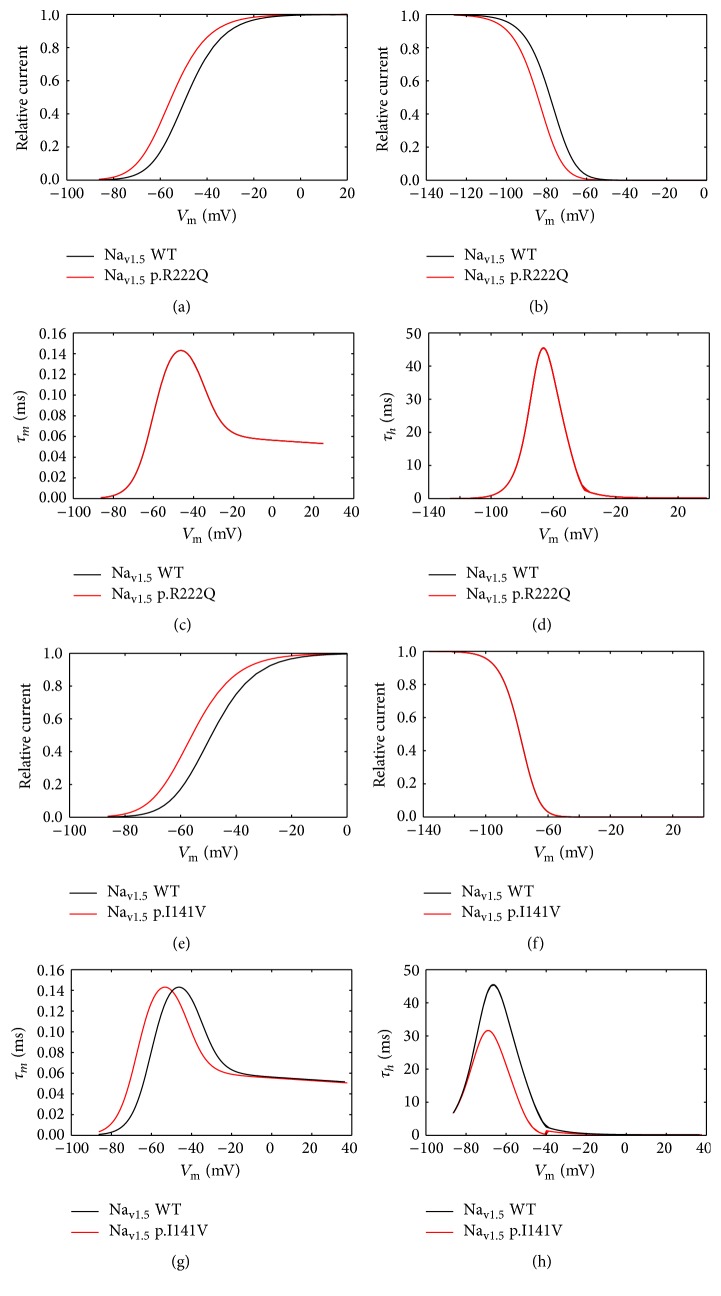
Effects of the p.R222Q and p.I141V mutations on *I*_Na_ properties in the human ventricular cell model (TNNP cell model). (a) and (b) Effect of the p.R222Q mutation on the voltage dependence of steady state of activation and inactivation. (c) and (d) Effect of the p.R222Q mutation on the activation and inactivation kinetics. (e) and (f) Effect of the p.I141V mutation on the voltage dependence of steady state of activation and inactivation. (g) and (h) Effect of the p.I141V mutation on the activation and inactivation kinetics. For all panels, black lines: WT condition; red lines: mutant conditions.

**Figure 7:**
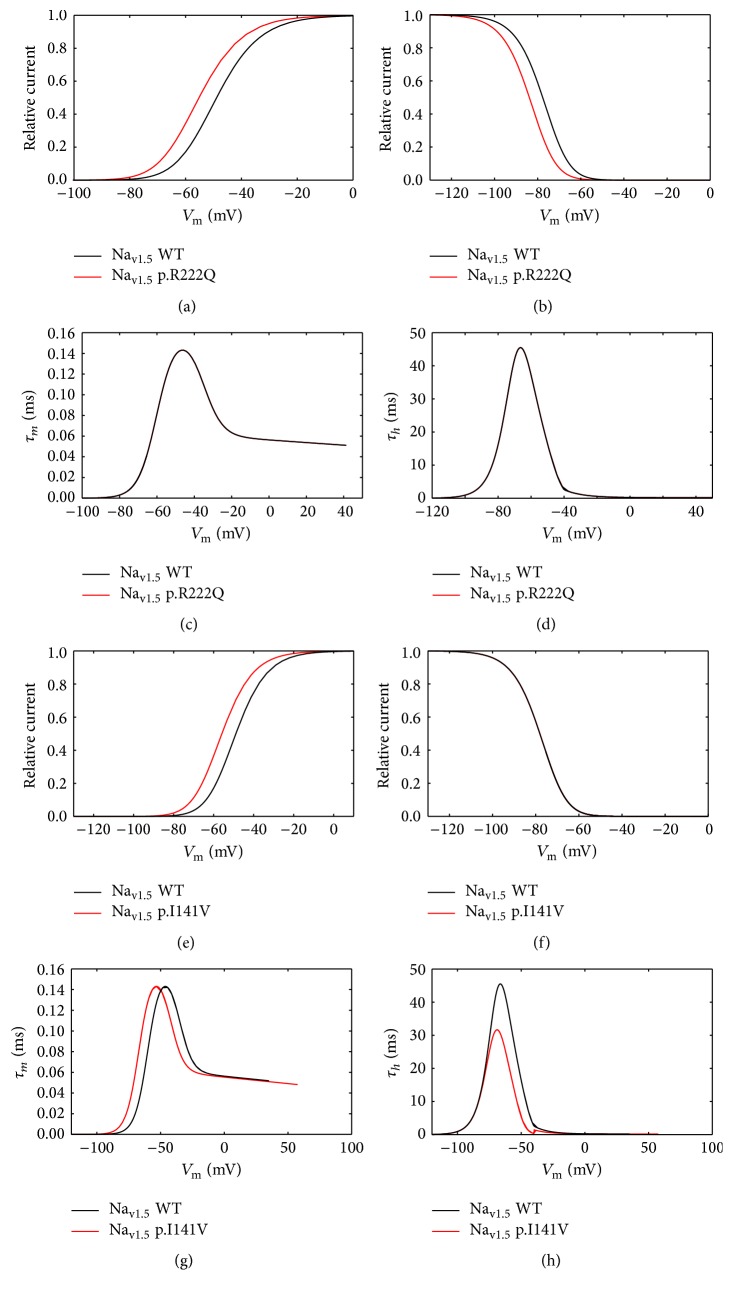
Effects of p.R222Q and p.I141V mutations on *I*_Na_ properties in the human Purkinje cell model (SANNBZ cell model). (a) and (b) Effect of the p.R222Q mutation on the voltage dependence of steady state of activation and inactivation. (c) and (d) Effect of the p.R222Q mutation on the activation and inactivation kinetics. (e) and (f) Effect of the p.I141V mutation on the voltage dependence of steady state of activation and inactivation. (g) and (h) Effect of the p.I141V mutation on the activation and inactivation kinetics. For all panels, black lines: WT condition; red lines: mutant conditions.

According to the experimental work of Kang et al. [8], the E3G antiarrhythmic effect, in the sitting of MEPPC and EPVT cardiac disorders, was evaluated. First, we incorporated the biophysical modifications that are induced by the Na_v1.5_-p.R222Q and Na_v1.5_-p.I141V mutations and got insight on their effects on atrial, ventricular, and Purkinje APs using MGTG, TNNP, and SANNBZ models (Figures 5, 6, and 7).

**Figure 8:**
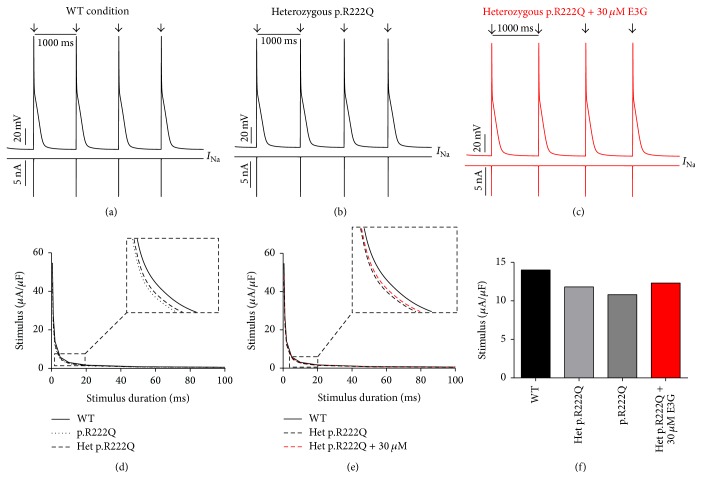
Effects of 30 *μ*M E3G on atrial cell action potentials for Na_v1.5_-WT and Na_v1.5_-p.R222Q conditions (MGTG cell model). (a), (b), and (c) Simulated APs (Top of the panels) and the cardiac sodium currents (Bottom of the panels) in WT, heterozygous Na_v1.5_-p.R222Q, and heterozygous Na_v1.5_-p.R222Q + 30 *μ*M conditions at 1 Hz cycle length. Arrows: external stimulus. (d) and (e) Strength-duration curves in the MGTG cell model for Na_v1.5_-WT and heterozygous Na_v1.5_-p.R222Q conditions with or without 30 *μ*M E3G (inset, zoom on the strength-duration curves). (f) Excitation thresholds at 2 ms stimulus duration in the MGTG atrial AP or without 30 *μ*M E3G.

**Figure 9 fig9:**
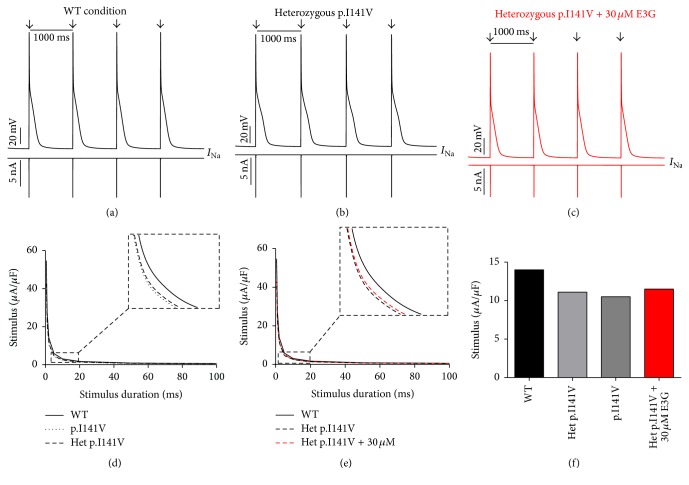
Effects of 30 *μ*M E3G on atrial cell action potentials for Na_v1.5_-WT and Na_v1.5_-p.I141V conditions (MGTG cell model). (a), (b), and (c) Simulated APs (Top of the panels) and the cardiac sodium currents (Bottom of the panels) in WT, heterozygous Na_v1.5_-p.I141V, and heterozygous Na_v1.5_-p.I141V + 30 *μ*M conditions at 1 Hz cycle length. Arrows: external stimulus. (d) and (e) Strength-duration curves in the MGTG cell model for Na_v1.5_-WT and heterozygous Na_v1.5_-p.I141V conditions with or without 30 *μ*M E3G (inset, zoom on the strength-duration curves). (f) Excitation thresholds at 2 ms stimulus duration in the MGTG atrial AP or without 30 *μ*M E3G.

**Figure 10 fig10:**
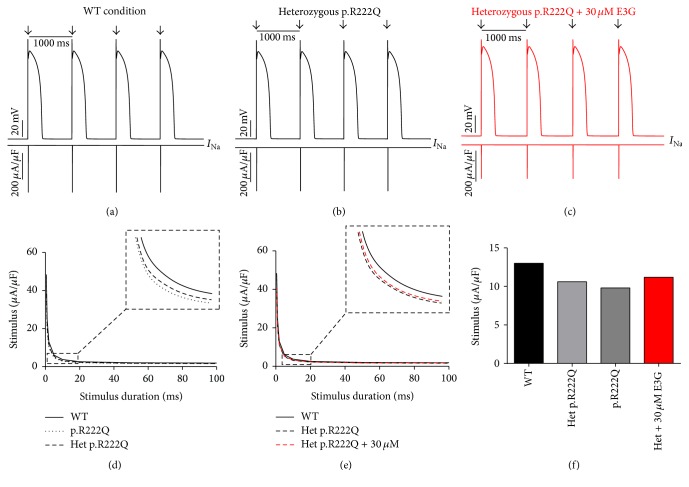
Effects of 30 *μ*M E3G on ventricular cell action potentials for Na_v1.5_-WT and Na_v1.5_-p.R222Q conditions (TNNP cell model). (a), (b), and (c) Simulated APs (Top of the panels) and the cardiac sodium currents (Bottom of the panels) in WT, heterozygous Na_v1.5_-p.R222Q, and heterozygous Na_v1.5_-p.R222Q + 30 *μ*M conditions at 1 Hz cycle length. Arrows: external stimulus. (d) and (e) Strength-duration curves in the TNNP cell model for Na_v1.5_-WT and heterozygous Na_v1.5_-p.R222Q conditions with or without 30 *μ*M E3G (inset, zoom on the strength-duration curves). (f) Excitation thresholds at 2 ms stimulus duration in the TNNP atrial AP or without 30 *μ*M E3G.

**Figure 11 fig11:**
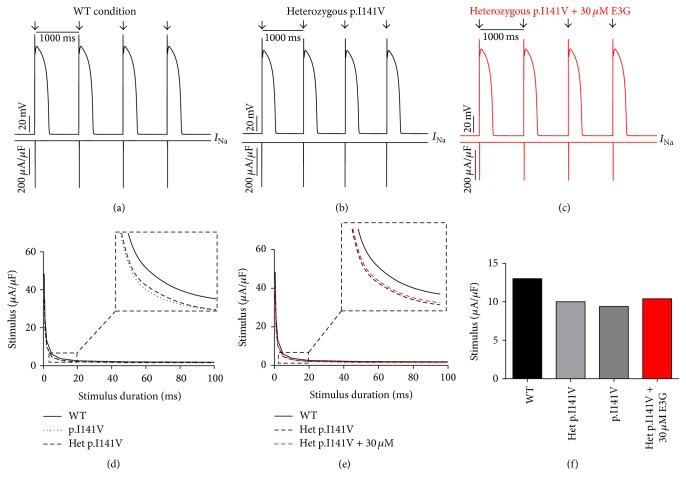
Effects of 30 *μ*M E3G on ventricular cell action potentials for Na_v1.5_-WT and Na_v1.5_-p.I141V conditions (TNNP cell model). (a), (b), and (c) Simulated APs (Top of the panels) and the cardiac sodium currents (Bottom of the panels) in WT, heterozygous Na_v1.5_-p.I141V, and heterozygous Na_v1.5_-p.I141V + 30 *μ*M conditions at 1 Hz cycle length. Arrows: external stimulus. (d) and (e) Strength-duration curves in the TNNP cell model for Na_v1.5_-WT and heterozygous Na_v1.5_-p.I141V conditions with or without 30 *μ*M E3G (inset, zoom on the strength-duration curves). (f) Excitation thresholds at 2 ms stimulus duration in the TNNP atrial AP or without 30 *μ*M E3G.

**Figure 12 fig12:**
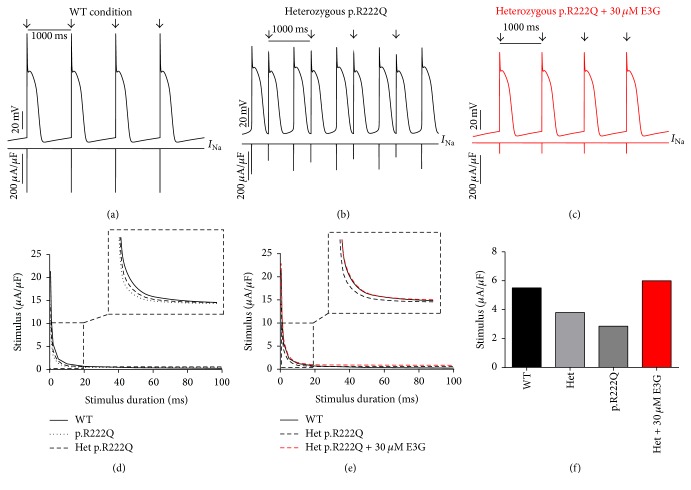
Effects of 30 *μ*M E3G on Purkinje cell action potentials for Na_v1.5_-WT and Na_v1.5_-p.R222Q conditions (SANNBZ cell model). (a), (b), and (c) Simulated APs (Top of the panels) and the cardiac sodium currents (Bottom of the panels) in WT, heterozygous Na_v1.5_- p.R222Q, and heterozygous Na_v1.5_-p.R222Q + 30 *μ*M conditions at 1 Hz cycle length. Arrows: external stimulus. (d) and (e) Strength-duration curves in the SANNBZ cell model for Na_v1.5_-WT and heterozygous Na_v1.5_-p.R222Q conditions with or without 30 *μ*M E3G at 2.5 Hz (inset, zoom on the strength-duration curves). (f) Excitation thresholds at 2 ms stimulus duration in the SANNBZ atrial AP or without 30 *μ*M E3G.

**Figure 13:**
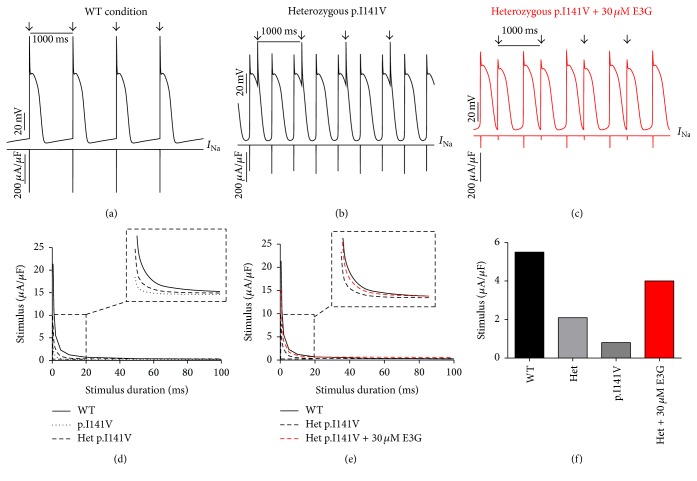
Effects of 30 *μ*M E3G on Purkinje cell action potentials for Na_v1.5_-WT and Na_v1.5_-p.I141V conditions (SANNBZ cell model). (a), (b), and (c) Simulated APs (Top of the panels) and the cardiac sodium currents (Bottom of the panels) in WT, heterozygous Na_v1.5_-p.I141V, and heterozygous Na_v1.5_-p.I141V + 30 *μ*M conditions at 1 Hz cycle length. Arrows: external stimulus. (d) and (e) Strength-duration curves in the SANNBZ cell model for Na_v1.5_-WT and heterozygous Na_v1.5_-p.I141V conditions with or without 30 *μ*M E3G at 2.5 Hz (inset, zoom on the strength-duration curves). (f) Excitation thresholds at 2 ms stimulus duration in the SANNBZ atrial AP or without 30 *μ*M E3G.

Interestingly, the introduction of equations mimicking the heterozygous state into the atrial and ventricular cell models induced minor changes in their AP morphologies (Figures 8(a), 8(b), 9(a), 9(b), 10(a), 10(b), 11(a), and 11(b)). Conversely, Mann et al. and Swan et al. [3, 4] reported a drastic effect when these equations are introduced in the Purkinje cell model. Indeed, for the Na_v1.5_-p.R222Q and Na_v1.5_-p.I141V mutants, the model showed an accelerated rate of spontaneous activity of Purkinje cells leading to the occurrence of ectopic beats during the diastolic interval at 1 Hz (Figures 12(a), 12(b), 13(a), and 13(b)). These abnormalities disappeared at higher pacing rates (Data not shown).

On the other hand, using MGTG, TNNP, and SANNBZ models, strength-duration curves were constructed. In the presence of Na_v1.5_-p.R222Q and Na_v1.5_-p.I141V mutations, a lower excitation threshold for action potential generation (pacing rates: 1 Hz for atrial and ventricular models and 2.5 Hz for the Purkinje model) was observed in the p.R222Q and p.I141V mutations in homozygous and heterozygous genotypes compared with the WT ((d) in Figures 8 [Fig fig9] [Fig fig10] [Fig fig11] [Fig fig12]–13).

Conduction velocity was investigated in fibers of MGTG, TNNP, and SANNBZ cell models (pacing rates, as described above). The presence of the Na_v1.5_-p.I141V mutation, in homozygous and heterozygous states, accelerated atrial and ventricular conduction at 1 Hz and Purkinje conduction at 2.5 Hz. Similar variations were observed in Na_v1.5_-p.R222Q mutation, whereas the conduction velocity was lower than WT condition in Purkinje cell model at 2.5 Hz pacing rate (Table 4).

Of note, strength-duration curves and conduction velocities could not be established at 1 Hz in the Purkinje model because of the accelerated spontaneous rhythm caused by p.R222Q and p.I141V mutations.

### 3.3. Antiarrhythmic Effect of E3G on SCN5A-Related Cardiac Syndrome, MEPPC, and EPVT

To investigate the potential antiarrhythmic effect of E3G on Na_v1.5_-p.R222Q and Na_v1.5_-p.I141V-related cardiac syndromes, the reported experimental effects of E3G were tested on these mutants. As shown in (a), (b), and (c) in Figures 8 [Fig fig9] [Fig fig10]–11, 30 *μ*M E3G does not affect the shape of action potentials in atrial and ventricular cells compared to the WT, Na_v1.5_-p.R222Q, and Na_v1.5_-p.I141V heterozygous conditions. However, this compound suppressed the ectopic APs observed in the presence of Na_v1.5_-p.R222Q mutation in Purkinje cell model (Figure 12(c)). Similar effects were obtained for Na_v1.5_-p.I141V mutation. Indeed, E3G decreased the number of ectopic beats associated with the presence of this mutation in Purkinje cells (Figure 13(c)). Of note, for these simulations, the models were stabilized during 60 s, then supplementary run was started for another 60 s, and finally the last 5 s of each supplementary run was analyzed.

Moreover, simulations were run using MGTG, TNNP, and SANNBZ cell models, and the strength-duration curves were constructed. In these models, the addition of 30 *μ*M E3G decreased the excitability of the atrial, ventricular, and Purkinje cells by increasing the excitation threshold for action potential generation ((e) and (f) in Figures 8 [Fig fig9] [Fig fig10] [Fig fig11] [Fig fig12]–13).

Finally, the effect of E3G on conduction velocity was investigated in the presence of p.R222Q and p.I141V mutations using fibers of atrial, ventricular, and Purkinje cell models. The presence of 30 *μ*M E3G attenuates the effect of p.R222Q and p.I141V mutations by decreasing atrial, ventricular, and Purkinje conductions. Of note, the conduction velocity was calculated at 1 Hz for the atrial and ventricular models and at 2.5 Hz for the Purkinje cells (Table 4).

## 4. Discussion

The aim of this study was to evaluate the benefits-risks balance of E3G consumption on the setting of cardiac channelopathies. Two gain-of-function mutations, Na_v1.5_-p.R222Q and Na_v1.5_-p.I141V, linked, respectively, with MEPPC and EPVT have been studied. Computer simulations of action potentials showed that 30 *μ*M E3G reduced the excitability of Purkinje cells for the EPVT and suppressed the AP abnormalities characteristic of the MEPPC phenotype.

### 4.1. Simulated Effect of E3G on the Electrophysiological Properties of Cardiac Cells

Epigallocatechin-3-Gallate is the major catechin found in green tea. Tested at a dose of 30 *μ*M, E3G modulates several voltage gated ion channels such as sodium, L-type calcium, and KCNQ1 channels [8]. Thus, the perfusion of this catechin induces several electrocardiographic modifications in Langendorff-perfused guinea pig hearts [8]. Indeed, E3G prolonged PR and QRS intervals, slightly shortened the QT interval, and altered the shape of the ST-T-wave segment [8]. To explain the link between these effects,* in silico* E3G effects were reproduced as described by Kang et al. [8]. Then, the effect of these modifications was evaluated on cardiac action potentials. Either for atrial, ventricular, or Purkinje cell models, our simulations showed delayed action potentials upstrokes, decreased APs amplitudes, and reduced conduction velocity in these cell models. These effects are likely related to the inhibition of the cardiac sodium channel by E3G. In fact, these parameters result mainly from the passage of sodium ions through these channels. Therefore, E3G effects are reflected on the whole heart activity by the prolongation of the PR and QRS intervals.

On the other hand, the experimental work of Kang et al. showed a slight decrease of the QT interval when E3G is perfused [8]. These findings are consistent with our results showing a slight diminution of ventricular AP duration in presence of E3G. However, our simulations failed to explain the increase of the interval from the peak of the T wave to the end of the T wave (T_p_-T_e_) on the resting guinea pig ECG. This interval reflects the transmural dispersion of repolarization in the ventricle [20, 21]. Accordingly, it was shown that the increase of this interval could be associated with an increase of the repolarization heterogeneity across the ventricular wall with an increased risk of sudden cardiac death [22]. In contrast to these evidences, our results predicted a slight decrease of this heterogeneity.

### 4.2. The p.R222Q and p.I141V Effects on Cardiac Excitability

In order to evaluate the possible antiarrhythmic effect of E3G in the sitting of MEPPC and EPVT cardiac disorders, the biophysical modifications induced by the Na_v1.5_-p.R222Q and Na_v1.5_-p.I141V mutants were incorporated as previously described by Mann et al. and Swan et al. [3, 4]. As shown by these groups, the introduction of these modifications into the atrial and ventricular cell models does not affect their AP morphologies. In contrast, the incorporation of the Na_v1.5_-p.R222Q and Na_v1.5_-p.I141V biophysical effects in the Purkinje cell model strongly affects the normal activation of these cells. Indeed, for these mutants, the model showed an accelerated rate of spontaneous activity of Purkinje cells leading to the occurrence of ectopic beats during the diastolic interval. In relevance to the disappearance of clinically observed premature contractions during exercise, the Purkinje ectopic APs induced by Na_v1.5_-p.R222Q mutation disappear at high pacing rates (Data not shown).

On the other hand, in atrial, ventricular, and Purkinje models, the presence of Na_v1.5_-p.R222Q and Na_v1.5_-p.I141V mutations lowers the excitation thresholds for action potential generation compared with the WT. Thus, threshold potential could be rapidly reached during the diastolic depolarization phase and consequently fire more action potentials in the Purkinje fiber compared to the WT condition. In contrast, atrial and ventricular cells do not show any spontaneous or ectopic activity in the presence of Na_v1.5_-p.R222Q and Na_v1.5_-p.I141V mutations. The biophysical modification induced by these mutations may promote the onset of arrhythmias by increasing the excitability of atrial and ventricular cells but cannot induce a spontaneous activation of these cells. Thus, as described by Laurent et al., the premature ventricular contraction observed in the affected patients may be triggered via the abnormal activity of the Purkinje fibers in the presence of p.R222Q and p.I141V mutations [2].

Moreover, the investigation of conduction velocity in fibers of MGTG, TNNP, and SANNBZ cell models shows an accelerated atrial, ventricular, and Purkinje conduction in the presence of Na_v1.5_-p.I141V mutation. Similar variations were observed in Na_v1.5_-p.R222Q mutation, whereas the conduction velocity was lower than WT condition in Purkinje cell model. The difference between the two mutations is related to the left shift of the steady state of inactivation shown by the Na_v1.5_-p.R222Q mutant. Indeed, when the Na_v1.5_-p.R222Q effect on the steady state of inactivation was suppressed, an increase of the conduction velocity was observed (Data not shown).

### 4.3. Antiarrhythmic Effect of E3G on the MEPPC and EPVT Syndromes

By implementing the experimental functional effects of E3G on the cardiac ion channels, we investigated whether E3G had an antiarrhythmic effect on Na_v1.5_-p.R222Q and Na_v1.5_-p.I141V-related cardiac syndromes.

Regarding the Na_v1.5_-p.R222Q mutation, the application of 30 *μ*M E3G suppressed the ectopic APs characteristic of the MEPPC phenotype. However, at the same dose, E3G partially reduced the frequency of ectopic beats observed in Purkinje cells for the EPVT. These effects could be related to the inhibitory action of this compound on cardiac sodium channels, as was described in some antiarrhythmic drugs such as Quinidine and flecainide [2, 23]. In addition, the increase of excitation threshold for action potential generation and the decrease of conduction velocities, in the presence of 30 *μ*M E3G, may also limit the cardiac cells hyperexcitability that is related to gain of functions mutations of the cardiac sodium channels.

On the other hand, a clear difference in E3G effect was observed between the MEPPC and EPVT disorders. This difference could be explained by the biophysical properties of Na_v1.5_-p.R222Q and Na_v1.5_-p.I141V mutants. Indeed, in contrast to the Na_v1.5_-p.I141V mutation, known by the sole modification of the activation process of Na_v1.5_, the Na_v1.5_-p.R222Q mutation shifted the activation and inactivation processes towards more negative potentials [2, 3]. Therefore, E3G generated a more pronounced loss of function in the Na_v1.5_-p.R222Q mutant than the Na_v1.5_-p.I141V one. This is presumably due to the magnification of the negative shifts of Na_v1.5_ steady state inactivation induced in presence of both E3G and Na_v1.5_-p.R222Q.

## 5. Conclusion

The present simulations suggest that E3G consumption may have a beneficial effect in the setting of cardiac sodium channelopathies displaying hyperexcitability phenotypes. Thus, this compound may offer a new promising alternative to prevent and treat the episodes of arrhythmia.

## Figures and Tables

**Table 1:** Formulation of WT and mutated sodium channels (Na_v1.5_-p.R222Q and Na_v1.5_-p.I141V) in the presence or absence of 30 *µ*M of E3G. The bold font corresponds to mutations effect and the bold-italic font to E3G effects.

	TNNP/SANNBZ models, p.R222Q.*I*_Na_	TNNP/SANNBZ models, p.I141V.*I*_Na_	TNNP/SANNBZ models, *I*_CaL_	TNNP/SANNB models, *I*_Ks_
WT	*m*_*∞*_, WT (*V*_m_) = *m*_*∞*_, WT (*V*_m_) *α*_*m*_, WT (*V*_m_) = *α*_*m*_, WT (*V*_m_) *β*_*m*_ WT (*V*_m_) = *β*_*m*_, WT (*V*_m_) *h*_*∞*_, WT (*V*_m_) = *h*_*∞*_, WT (*V*_m_) *β*_*h*_WT (*V*_m_) = *β*_*m*_, WT (*V*_m_)	*m*_*∞*_, WT (*V*_m_) = *m*_*∞*_, WT (*V*_m_) *α*_*m*_, WT (*V*_m_) = *α*_*m*_, WT (*V*_m_) *β*_*m*_ WT (*V*_m_) = *β*_*m*_, WT (*V*_m_) *h*_*∞*_, WT (*V*_m_) = *h*_*∞*_, WT (*V*_m_) *β*_*h*_ WT (*V*_m_) = *β*_*m*_, WT (*V*_m_)	100% of *I*_CaL_	100% of *I*_Ks_

WT + 30 *µ*M E3G	*m*_*∞*_, WT (*V*_m_) = *m*_*∞*_, WT (*V*_m_) *α*_*m*_, WT (*V*_m_) = *α*_*m*_, WT (*V*_m_) *β*_*m*_ WT (*V*_m_) = *β*_*m*_, WT (*V*_m_) *h*_*∞*_, WT (*V*_m_) = *h*_*∞*_, WT (*V*_m_ + ***6***) *β*_*h*_WT (*V*_m_) = *β*_*h*_, WT (*V*_m_)	*m*_*∞*_, WT (*V*_m_) = *m*_*∞*_, WT (*V*_m_) *α*_*m*_, WT (*V*_m_) = *α*_*m*_, WT (*V*_m_) *β*_*m*_ WT (*V*_m_) = *β*_*m*_, WT (*V*_m_) *h*_*∞*_, WT (*V*_m_) = *h*_*∞*_, WT (*V*_m_ + ***6***) *β*_*h*_WT (*V*_m_) = *β*_*h*_, WT (*V*_m_)	***80%*** of *I*_CaL_	***50%*** of *I*_Ks_

Mutants	*m*_*∞*_, p.I141V (*V*_m_) = *m*_*∞*_, WT (*V*_m_ − 6.3)	*m*_*∞*_, p.I141V (*V*_m_) = *m*_*∞*_, WT (*V*_m_ − 7)	100% of *I*_CaL_	100% of *I*_Ks_
	*α*_*m*_, p.I141V (*V*_m_) = *α*_*h*_, WT (*V*_m_)	*α*_*m*_, p.I141V (*V*_m_) = *α*_*h*_, WT (*V*_m_ − 7)		
	*β*_*m*_, p.I141V (*V*_m_) = *β*_*h*_, WT (*V*_m_)	*β*_*m*_, p.I141V (*V*_m_) = *β*_*h*_, WT (*V*_m_ + **7**)		
	*h*_*∞*_, p.I141V (*V*_m_) = *h*_*∞*_, WT (*V*_m_ + **6.2**) *β*_*h*_WT (*V*_m_) = *β*_*h*_, WT (*V*_m_)	*h*_*∞*_, p.I141V (*V*_m_) = *h*_*∞*_, WT (*V*_m_) *β*_*h*_WT (*V*_m_) = *β*_*h*_, WT (*V*_m_ + **7**)		

Mutants + 30 *µ*M E3G	*m*_*∞*_, p.I141V (*V*_m_) = *m*_*∞*_, WT (*V*_m_ − 6.3)	*m*_*∞*_, p.I141V (*V*_m_) = *m*_*∞*_, WT (*V*_m_ − 7)	***80%*** of *I*_CaL_	***50%*** of *I*_Ks_
	*α*_*m*_, p.I141V (*V*_m_) = *α*_*h*_, WT (*V*_m_)	*α*_*m*_, p.I141V (*V*_m_) = *α*_*h*_, WT (*V*_m_ − 7)		
	*β*_*m*_, p.I141V (*V*_m_) = *β*_*h*_, WT (*V*_m_)	*β*_*m*_, p.I141V (*V*_m_) = *β*_*h*_, WT (*V*_m_ + **7**)		
	*h*_*∞*_, p.I141V (*V*_m_) = *h*_*∞*_, WT (*V*_m_ + **6.2** + ***6***)	*h*_*∞*_, p.I141V (*V*_m_) = *h*_*∞*_, WT (*V*_m_ + ***6***)		
	*β*_*h*_WT (*V*_m_) = *β*_*h*_, WT (*V*_m_)	*β*_*h*_WT (*V*_m_) = *β*_*h*_, WT (*V*_m_ + **7**)		

**Table 2:** Formulation of WT and mutated sodium channels (Na_v1.5_-p.R222Q and Na_v1.5_-p.I141V) in the presence or absence of 30 *µ*M of E3G in the Maleckar-Greenstein-Trayanova-Giles atrial cell model. The bold font corresponds to mutations effect and the bold-italic font to E3G effects.

	MGTG atrial model, p.R222Q.*I*_Na_	MGTG atrial model, p.I141V.*I*_Na_	MGTG atrial model, *I*_CaL_	MGTG atrial model, *I*_Ks_
* *WT	*m*_*∞*_, WT (*V*_m_) = *m*_*∞*_, WT (*V*_m_)	*m*_*∞*_, WT (*V*_m_) = *m*_*∞*_, WT (*V*_m_)	100% of *I*_CaL_	100% of *I*_Ks_
	*m* factor, WT (*V*_m_) = *m* factor, WT (*V*_m_) *τ*_*m*_ = 4.2*e* − 5 (s)*∗*exp⁡(−*m*_factor_ *∗m*_factor_) + 2.4*e* − 5 (s)	*m* factor, WT (*V*_m_) = *m* factor, WT (*V*_m_) *τ*_*m*_ = 4.2*e* − 5 (s)*∗*exp⁡(−*m*_factor_ *∗m*_factor_) + 2.4*e* − 5 (s)		
	*h*_*∞*_, WT (*V*_m_) = *h*_*∞*_, WT (*V*_m_)	*h*_*∞*_, WT (*V*_m_) = *h*_*∞*_, WT (*V*_m_)		
	*h* factor, WT (*V*_m_) = *h* factor, WT (*V*_m_)	*h* factor, WT (*V*_m_) = *h* factor, WT (*V*_m_)		
	*τ*_*h*1_ = 0.03 (s) *∗h*_factor_ + 0.0003 (s)	*τ*_*h*1_ = 0.03 (s) *∗* *h*_factor_ + 0.0003 (s)		

WT + 30 *µ*M E3G	*m*_*∞*_, WT (*V*_m_) = *m*_*∞*_, WT (*V*_m_)	*m*_*∞*_, WT (*V*_m_) = *m*_*∞*_, WT (*V*_m_)	***80%*** of *I*_CaL_	***50%*** of *I*_Ks_
	*m* factor, WT (*V*_m_) = *m* factor, WT (*V*_m_)	*m* factor, WT (*V*_m_) = *m* factor, WT (*V*_m_)		
	*τ*_*m*_ = 4.2*e* − 5 (s)*∗*exp⁡(−*m*_factor_ *∗m*_factor_) + 2.4*e* − 5 (s)	*τ*_*m*_ = 4.2*e* − 5 (s)*∗*exp⁡(−*m*_factor_ *∗m*_factor_) + 2.4*e* − 5 (s)		
	*h*_*∞*_, WT (*V*_m_) = *h*_*∞*_, WT (*V*_m_ + ***6***)	*h*_*∞*_, WT (*V*_m_) = *h*_*∞*_, WT (*V*_m_ + ***6***)		
	*h* factor, WT (*V*_m_) = *h* factor, WT (*V*_m_)	*h* factor, WT (*V*_m_) = *h* factor, WT (*V*_m_)		
	*τ*_*h*1_ = 0.03 (s) *∗* *h*_factor_ + 0.0003 (s)	*τ*_*h*1_ = 0.03 (s) *∗* *h*_factor_ + 0.0003 (s)		

Mutants	*m*_*∞*_, WT (*V*_m_) = *m*_∞_, WT (*V*_m_ + **6.3**)	*m*_*∞*_, WT (*V*_m_) = *m*_*∞*_, WT (*V*_m_ + **7**)	100% of *I*_CaL_	100% of *I*_Ks_
	*m* factor, WT (*V*_m_) = *m* factor, WT (*V*_m_)	*m* factor, WT (*V*_m_) = *m* factor, WT (*V*_m_ + **7**)		
	*τ*_*m*_ = 4.2*e* − 5 (s)*∗*exp⁡(−*m*_factor_ *∗m*_factor_) + 2.4*e* − 5 (s)	*τ*_*m*_ = 4.2*e* − 5 (s)*∗*exp⁡(−*m*_factor_ *∗m*_factor_) + 2.4*e* − 5 (s)		
	*h*_*∞*_, WT (*V*_m_) = *h*_*∞*_, WT (*V*_m_ + **6.2**)	*h*_*∞*_, WT (*V*_m_) = *h*_*∞*_, WT (*V*_m_)		
	*h* factor, WT (*V*_m_) = *h* factor, WT (*V*_m_)	*h* factor, WT (*V*_m_) = *h* factor, WT (*V*_m_ + **7**)		
	*τ*_*h*1_ = 0.03 (s) *∗* *h*_factor_ + 0.0003 (s)	*τ*_*h*1_ = 0.03 (s) *∗* *h*_factor_ + 0.0003 (s)		

Mutants + 30 *µ*M E3G	*m*_*∞*_, WT (*V*_m_) = *m*_*∞*_, WT (*V*_m_ + **6.3**)	*m*_*∞*_, WT (*V*_m_) = *m*_*∞*_, WT (*V*_m_ + **7**)	***80%*** of *I*_CaL_	***50%*** of *I*_Ks_
	*m* factor, WT (*V*_m_) = *m* factor, WT (*V*_m_)	*m* factor, WT (*V*_m_) = *m* factor, WT (*V*_m_ + **7**)		
	*τ*_*m*_ = 4.2*e* − 5 (s)*∗*exp⁡(−*m*_factor_ *∗m*_factor_) + 2.4*e* − 5 (s)	*τ*_*m*_ = 4.2*e* − 5 (s)*∗*exp⁡(−*m*_factor_ *∗m*_factor_) + 2.4*e* − 5 (s)		
	*h*_*∞*_, WT (*V*_m_) = *h*_*∞*_, WT (*V*_m_ + **6.2** + ***6***)	*h*_*∞*_, WT (*V*_m_) = *h*_*∞*_, WT (*V*_m_ + ***6***)		
	*h* factor, WT (*V*_m_) = *h* factor, WT (*V*_m_)	*h* factor, WT (*V*_m_) = *h* factor, WT (*V*_m_ + **7**)		
	*τ*_*h*1_ = 0.03 (s) *∗* *h*_factor_ + 0.0003 (s)	*τ*_*h*1_ = 0.03 (s) *∗* *h*_factor_ + 0.0003 (s)		

**Table 3:** A summary of the parameters used for the calculation of conduction velocity in the atrial, ventricular, and Purkinje models.

Model	Cell number (*n*)	Intercellular conductance (mS/*µ*F)	Step size (ms)
MGTG	100	17	0.01
SANNBZ	100	17	0.01
TNNP	100	7	0.001

**Table 4:** The effect of E3G on conduction velocity investigations in the presence of p.R222Q and p.I141V in heterozygous states.

Model	Atrial cells		Ventricular cells		Purkinje cells	
Condition	CV (cm/s) control	CV (cm/s) 30 *µ*M E3G	CV (cm/s) control	CV (cm/s) 30 *µ*M E3G	CV (cm/s) control	CV (cm/s) 30 *µ*M E3G
Na_v1.5_ WT	55.07	50.84	49.91	45.72	67.68	37.20
Na_v1.5_ p.I141V	57.44	53.93	53.63	49.11	72.43	42. 92
Na_v1.5_ p.R222Q	60.93	56.23	51.93	45.56	62.72	—

## Additional Points

*Limitations of the Study*. The current study has several limitations: (i) the used cellular models did not incorporate the effects of adrenergic stimulation. (ii) Any biological variability was incorporated in these simulations. (iii) We assumed that WT and mutants ions channels have similar affinity to E3G. (iv) Kinetic effects of E3G were not considered in this study. (v) Cell-level arrhythmias such as early after depolarization and delayed after depolarization, or reentry, are not investigated in the current study. (vi) Any comparison of E3G effects was realized in the used action potential models.
